# Supplementary material for: Nearest vent, dearest friend: biodiversity of Tiancheng vent field reveals cross-ridge similarities in the Indian Ocean
Source: R Soc Open Sci. 2020 Mar 25;7(3):200110. doi: 10.1098/rsos.200110 (PMC7137978; doi:10.1098/rsos.200110)
Supplement: Supplementary materials [file rsos200110supp1.docx]

**Supplementary figure S1** the location of three venting sites within the Tiancheng vent field.

**­­
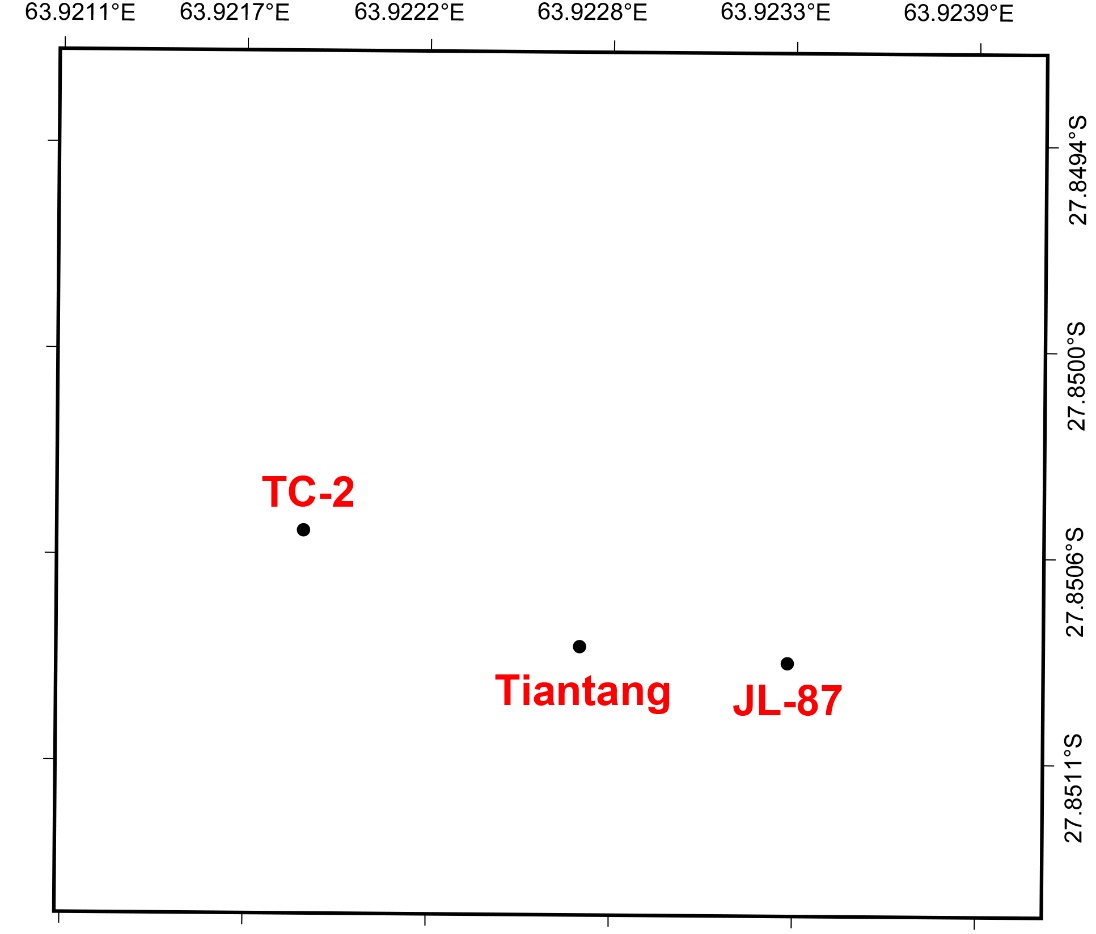
**

**Supplementary figure S2** EDS net counts of comparable area for the outer mineralised layer and scale organic matrix in the Tiancheng scaly-foot snail.**
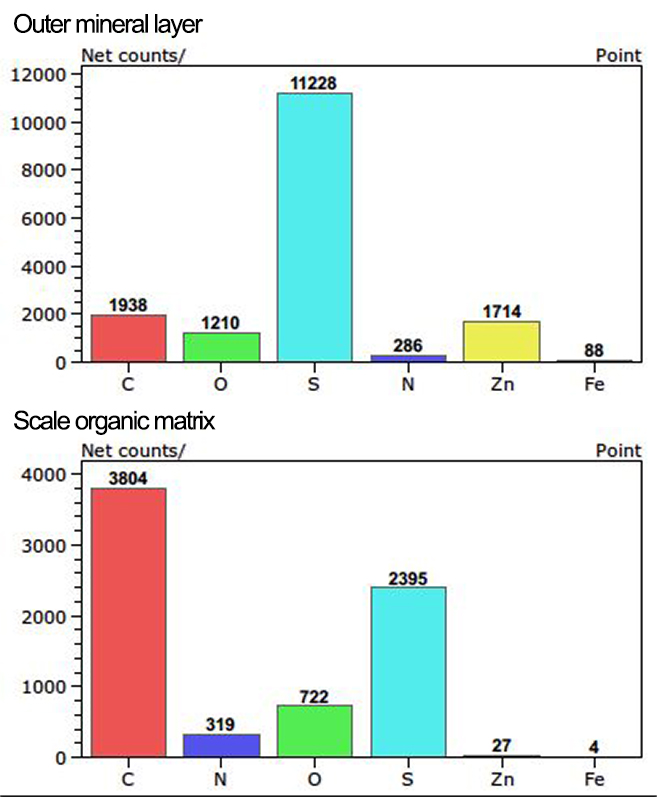
**

**Table S1a.** Summary table of Pairwise *F_s_*_t_ value of three *Austinograea rodriguezensis* populations in the Indian Ocean

|  | **Kairei** | **Solitaire** | **Tiancheng** |
| --- | --- | --- | --- |
| **Kairei** |  |  |  |
| **Solitaire** | 0.00066 |  |  |
| **Tiancheng** | -0.00048 | -0.00707 |  |

**Table S1b.** A Summary table of Pairwise *F_st_* value of five *Bathymodiolus marisindicus* populations in the Indian Ocean

|  | **Kairei** | **Tiancheng** | **Longqi** | **Edmond** | **Duanqiao** |
| --- | --- | --- | --- | --- | --- |
| **Kairei** |  |  |  |  |  |
| **Tiancheng** | -0.01287 |  |  |  |  |
| **Longqi** | -0.01924 | -0.02704 |  |  |  |
| **Edmond** | 0.00356 | -0.01301 | -0.01937 |  |  |
| **Duanqiao** | 0.02680* | -0.00400 | 0.00014 | -0.00340 |  |

**p*-value < 0.05

**Table S2a** Summary of genetic diversity of *Austinograea rodriguezensis*

|  | **Number of individuals** | **Number of Haplotypes** | **Haplotypes diversity** | **Nucleotide diversity** |
| --- | --- | --- | --- | --- |
| **Kairei** | 13 | 9 | 0.910 ± 0.0683 | 0.00282 ± 0.00186 |
| **Solitaire** | 14 | 7 | 0.872 ± 0.067 | 0.00314 ± 0.00203 |
| **Tiancheng** | 15 | 8 | 0.886 ± 0.054 | 0.00228 ± 0.00037 |

**Table S2b** Summary of genetic diversity of *Chrysomallon squamiferum*

|  | **Number of individuals** | **Number of Haplotypes** | **Haplotypes diversity** | **Nucleotide diversity** |
| --- | --- | --- | --- | --- |
| **Kairei** | 35 | 11 | 0.985 ± 0.0400 | 0.00380 ± 0.00220 |
| **Solitaire** | 23 | 14 | 0.971 ± 0.0320 | 0.00210 ± 0.00127 |
| **Tiancheng** | 20 | 10 | 0.858 ± 0.0620 | 0.00224 ± 0.00133 |
| **Longqi** | 35 | 8 | 0.700 ± 0.109 | 0.00344 ± 0.00194 |

**Table S2c** Summary Table of genetic diversity of *Bathymodiolus marisindicus*

|  | **Number of individuals** | **Number of Haplotypes** | **Haplotypes diversity** | **Nucleotide diversity** |
| --- | --- | --- | --- | --- |
| **Kairei** | 28 | 15 | 0.847 ± 0.066 | 0.00471 ± 0.00089 |
| **Tiancheng** | 19 | 9 | 0.772 ± 0.094 | 0.00608 ± 0.00160 |
| **Longqi** | 15 | 11 | 0.933 ± 0.054 | 0.00713 ± 0.00145 |
| **Edmond** | 29 | 19 | 0.906 ± 0.048 | 0.00664 ± 0.00107 |
| **Duanqiao** | 10 | 10 | 1.00 ± 0.045 | 0.01041 ± 0.00143 |
